# Migraine and fibromyalgia

**DOI:** 10.1186/1129-2377-16-S1-A45

**Published:** 2015-09-28

**Authors:** Marina de Tommaso

**Affiliations:** Basic Medical Neuroscience and Sensory System Department, Bari Aldo Moro University, Bari, Italy

Fibromyalgia is a chronic pain syndrome of unknown etiology characterized by diffuse pain, sleep disorders, fatigue, cognitive dysfunction and a cohort of different symptoms implying comorbidity with diseases with common pathophysiological basis. There is a growing body of evidence that abnormal pain processing at a central level has a role in FM pathogenesis, though recent evidence supports the coexistence of a peripheral nociceptive fibers sufferance. In recent years, clear phenomena of temporal summation of pain (or windup) and central sensitization have been extensively reported. Neurophysiologic methods able to explore the nociceptive afferent system suggest that FM syndrome is heterogeneous, with pain processing dysfunction at both peripheral and central level. Reduced habituation to multimodal and especially painful stimuli characterizes FM, as well as associated conditions, one of the most common is migraine. A genetic dysfunction of ionic channels may possibly explain neuronal abnormalities at both central and peripheral level in FM, opening a new scenario also in the comprehension of pathophysiological basis of associated conditions.

